# Rice physical defenses and their role against insect herbivores

**DOI:** 10.1007/s00425-024-04381-7

**Published:** 2024-04-02

**Authors:** Devi Balakrishnan, Nick Bateman, Rupesh R. Kariyat

**Affiliations:** Department of Entomology and Plant Pathology, University of Arkansas, Fayetteville, AR 72701 USA

**Keywords:** *Oryza sativa*, Epicuticular wax, Trichomes, Silicon, Callose, Lignin

## Abstract

*****Main conclusion***:**

**Understanding surface defenses, a relatively unexplored area in rice can provide valuable insight into constitutive and induced defenses against herbivores.**

**Abstract:**

Plants have evolved a multi-layered defense system against the wide range of pests that constantly attack them. Physical defenses comprised of trichomes, wax, silica, callose, and lignin, and are considered as the first line of defense against herbivory that can directly affect herbivores by restricting or deterring them. Most studies on physical defenses against insect herbivores have been focused on dicots compared to monocots, although monocots include one of the most important crops, rice, which half of the global population is dependent on as their staple food. In rice, Silica is an important element stimulating plant growth, although Silica has also been found to impart resistance against herbivores. However, other physical defenses in rice including wax, trichomes, callose, and lignin are less explored. A detailed exploration of the morphological structures and functional consequences of physical defense structures in rice can assist in incorporating these resistance traits in plant breeding and genetic improvement programs, and thereby potentially reduce the use of chemicals in the field. This mini review addresses these points with a closer look at current literature and prospects on rice physical defenses.

## Introduction

Rice, *Oryza sativa* L. (Family: Gramineae) is the major staple food crop cultivated and consumed by more than half of the global population (Sharif et al. 2014). Rice is the third most important crop after sugarcane and maize, in terms of production and is cultivated in more than 100 countries with an annual production of nearly 510 million tons of milled rice across 165 million hectares, with significant contributions from China, India, Indonesia, Bangladesh, and Vietnam. Major rice exporters include India, Thailand, Vietnam, and Pakistan, and are mainly imported to the Sub-Saharan Africa region accounting for 31% of the overall global imports (USDA 2022). According to FAO (2018), the five major importers of rice are China, Nigeria, the Islamic Republic of Iran, Saudi Arabia, and the Philippines—expected to be one-third of the global rice imports by 2027. Clearly, rice production is critical for food security on planet Earth. However, like most cultivated crops, rice also suffers from multiple stressors under different production systems across the world.

Stress in plants can be defined as the external conditions that negatively impact a plant's growth, development, or yield (Verma et al. 2014) and can be categorized as abiotic or biotic stress. Abiotic stress is caused by the environment and can be either physical or chemical, whereas biotic stress is caused by living organisms, such as viruses, bacteria, fungi, nematodes, insects (Table 1), and weeds. To avoid the selective pressure imposed by these stresses, plants have developed incredible defense mechanisms. Plant defenses can be either direct or indirect (Karban and Baldwin 1997; Howe and Jander 2008). Direct defenses are types of defenses that exhibit a pernicious effect on herbivores affecting their mobility, feeding, growth, and development (Kessler and Baldwin 2001). On the other hand, indirect defense is the attraction of natural enemies toward the plants by the volatiles released during the attack i.e., herbivore-induced plant volatiles (HIPV) (Kessler and Baldwin 2002; Arimura et al. 2005; Howe and Jander 2008Kariyat et al. 2012) or through extra floral nectaries (Rosumek et al.^2009^; Heil 2015) that is found to be increased/ induced by insect herbivory and initiate more parasitism and predation (Jones et al. 2017). Although plant defenses and insect counter defenses have been meticulously examined, most of these studies have ignored the first line of defense—plant surface defenses and focused more on the chemical defenses (Qi et al. 2018; Lu et al. 2018; Shi et al. 2019; Kariyat et al. 2017). In dicots, extensive research has been conducted to understand their defense systems against herbivorous attacks, particularly in model plants such as Arabidopsis (*Arabidopsis thaliana* (L.) Heynh.) and tomato (*Lycopersicon esculentum* (Mill.) (Wu and Baldwin 2010; Stam et al. 2013; Wang and Wu 2013), and in a wide range of wild and domesticated, model, and non-model species (Philipe and Bohlmann 2007; Xing et al. 2017; Feng et al. 2021; Johnson et al. 2021; Lefebvre et al. 2022; Kaur and Kariyat 2023). However, monocots, an important group that includes most of the staple food crops including rice, have been less studied, in physical and structural defenses. Here, we review and summarize the previous studies on physical defenses, their mode of action, and then specifically evaluate these defenses in rice, and suggest potential areas for future research.

**Table 1:** Major pests of rice and their taxonomic status

Scientific name	Common name	Order	Family	References
*Recilia dorsalis*	Zigzag leafhoppers	Hemiptera	Cicadellidae	Wilson and Claridge (1991); Chowdhury et al (2011)
*Nilaparvata lugens*	Brown Plant hopper		Delphacidae	Sogawa, (1982); Heinrichs and Mochida (1984); Backus et al. (2005)
*Cicadella viridis*	Green leaf hopper		Cicadellidae	Chu and Teng (1950)
*Scotinophara coaractata*	Rice black bug		Pentatomidae	Corbett and Yusope (1924); Barrion et al. (1982)
*Brevennia rehi*	Mealy bug		Pseudococcidae	Williams et al. (1981); Mishra et al. (2019)
*Oebalus pugnax*	Rice stink bug		Pentatomidae	Swanson and Newsom (1962); Bowling (1979), Way (2003); Patel et al. (2006), Awuni et al. (2015)
*Hysteroneura setariae*	Root aphid		Aphididae	Akinlosotu (1977); Nasruddin (2013)
*Pelopidas mathias*	Rice skipper	Lepidoptera	Hesperiidae	Teotia and Nand (1966); Litsinger et al. (1994a, b)
*Scirpophaga incertulas*	Yellow Stem borer		Pyraustidae	Catling et al. (1987); Bandong and Litsinger (2005)
*Spodoptera frugiperda*	Fall Army worm		Noctuidae	Pashley et al. (1987); Ashley et al. (1989); Nagoshi et al. (2021)
*Cnaphalocrocis medinalis*	Rice leaf folder		Crambidae	Rajamma and Das (1969); Maragesan and Chellish (1987); Khan et al. (1989); Kraker et al. (1999); Alvi et al.(2003)
*Melanitis ismene*	Green horned caterpillar		Sphingidae	Sajjan and Singh (1972); Chander (1998)
*Nymphula depunctalis*	Rice case worm		Pyralidae	Heinrichs and Viajante (1987); Litsinger et al. (1994a, b);
*Baliothrips biformis*	Rice thrips	Thysanoptera	Thripidae	Nugaliyadde and Heinrichs (1984); Velusamy (1990)
*Gryllus spp.*	Field cricket	Orthoptera	Gryllidae	Pathak and Khan (1994)
*Caelifera spp.*	Grasshopper		Acrididae, Tettigoniidae	Anne and Hussain, (2016); Mitku et al (2021)
*Lissorhoptrus oryzophilus*	Rice water weevil	Coleoptera	Curculionidae	Zou et al. (2004); Aghaee and Godfrey (2014); Mulcahy et al (2022)
*Dicladispa armigera*	Rice hispa		Chrysomelidae	Sen and Chakravorty (1970); Nath and Dutta (1997)
*Hydrellia spp.*	Rice whorl maggot	Diptera	Ephydridae	Viajante and Heinrichs (1986); Shepard et al (1990); Mangal (2000);
*Orseolia oryzae*	Rice gall midge		Cecidomyiidae	Heinrichs and Pathak (1980); Kumar et al. (2009)

## Physical defenses and their role against insect herbivores

Physical or structural barriers, which are often regarded as the first line of protection against herbivory, are direct defenses that include cuticle, wax, spines, trichomes, and thickening or lignification of the cell wall. These are structural modifications in plants that negatively impact herbivores and work in a distinct way to contribute to the integrated defense phenotype of plants. Interestingly, most of these defenses have been evolved against abiotic stressors, later diversified to be a major defense against biotic stressors (Kaur and Kariyat 2020). Among the structural barriers, the cuticle is the outermost layer composed of lipophilic compounds, laid over the epidermis protecting the plants from both biotic and abiotic stresses (Hanley et al. 2007; Agrawal et al. 2009). One of the most vital components of the cuticle is wax, the outermost protective barrier, and those dispersed on the surface of lipophilic polymer are called epicuticular wax (EW) (Wójcicka 2015). Wax can alter the feeding, movement, and foraging behavior of insect pests, predators, and parasitoids (Eigenbrode 2004), and the effects are species specific. For instance, in crucifers, the presence of long-chain alcohols and amyrins in the leaves reduced the infestation of the destructive diamondback moth, *Plutella xylostella* L. (Eigenbrode and Pillai 1998). Similarly, Watts and Kariyat (2022) studied the effects of epicuticular wax on tobacco hornworm, *Manduca sexta* by comparing two different species of Solanum (*Solanum glaucescens* Zuccarini and *Solanum macrocarpon* Linnaeus), the former with the highest wax and least trichomes, and latter with no wax and highest trichome density. They found epicuticular wax in *S. glaucescens* can act as a powerful barrier against *M. sexta* leading to the reduction in mass gain and increased mortality even when the species has the least trichomes. However, the role varies depending on the pests as in pea, *Pisum sativum* L., where fewer wax blossoms resulted in a reduction in aphid infestation but more severe weevil damage. (White and Eigenbrode 2000). Spines are another surface defense structure that are sharp, needle-like modifications of petioles, midrib, or spicules that disrupt the feeding, mobility, dispersal, and mating behavior of herbivores (Hanley et al. 2007; Portman et al. 2015; Kariyat et al. 2017). In Solanaceae, *Manduca sexta* preferred to defoliate plants with fewer spines, and opted quickly for leaves with no spines that are removed for the experiments compared to leaves with intact spines, confirming the role of spines in defense (Kariyat et al. 2017). Similarly in 2023, Johnson et al. investigated the effects of epicuticular wax of, *Aloe barbadensis* against *M. sexta* and *S. frugiperda* and found the surface waxes and volatiles emitted from the wax when added on artificial diet prevented them from feeding, also concluding the effects of wax affecting the growth and development of insect herbivores.

When compared to other surface barriers, trichomes are important, have undergone extensive research, and play a vital role in plant–biotic interactions (Kariyat et al. 2013, 2017). Trichomes are unicellular or multicellular hair-like appendages that advance outward (Werker 2000). They originate from the epidermal cells of vegetative and reproductive plant structures (Oksanen 2018) and have a negative impact on herbivores by preventing or impeding their movement or by delivering toxins that affect their growth and development (Agren and Schemske 1993; Kaur and Kariyat 2020). In addition to protecting against herbivores, they also provide defense against abiotic stresses such as UV radiation, water loss, and extreme temperatures (Ehleringer 1982; Li et al. 2018; Oksanen 2018). They can be divided broadly into glandular and non-glandular types (Werker 2000). Non-glandular trichomes are unicellular, tough, and sharp, blocking the entry or causing physical injury to insects (Dalin et al. 2008) and vary in their density, length, and orientation in different plant species (Cho et al. 2017; Kariyat et al. 2017, 2019; Watts and Kariyat 2021) whereas glandular trichomes are multicellular structures that trigger the genes that protect against insect herbivores by releasing toxic substances (Peiffer et al. 2009).

Lignification or cell wall thickening is another defense mechanism plants adopt in response to herbivory. Lignin is one of the most important phenolic acids and is the second most abundant polymer after cellulose, synthesized by phenylpropanoid pathway. Lignin is present in the cell wall of plants, imparting resistance to biotic and abiotic stresses in addition to structural support. Lignin is responsible for the toughness of tissue that can resist herbivore damage (Raupp 1985), -the tougher the tissue, the more the lignin is. In maize, lignin reduced the palatability to chewing insects by regulating the lignification process (Santiago et al. 2013) and by the effect of phenoloxidase enzymes that are involved in the lignin biosynthesis process. Phenoloxidase enzymes has also an important role in the production of toxic byproducts such as reactive oxygen species, peroxides, and quinones that are detrimental to herbivores (Felton et al. 1989; Gandhi et al. 2021). In general, the physical defenses in the plants have a great potential to tolerate herbivory and unveiling the role of each defense in depth can be used to modify and breed plants by regulating host plant resistance to reduce the attack from insect pests.

## Physical defenses in rice

### Epicuticular wax

In rice, epicuticular wax (EW) has been found to play an important role in the defense against herbivores. Brown plant hopper (BPH), *Nilaparvata lugens* (Stal)*,* a major sucking pest, has been found to alter its preference for the host by the presence of hydrocarbons and carbonyl groups in the wax. Woodhead and Pudgham (1988) investigated the non-preference of BPH on to the stem of variety IR46 compared to other varieties and their tendency to move from stem to the leaves of the variety, even though they prefer stems, thus concluding the role of wax as a structural barrier. The importance of EWs in the reduction of infestations of two important pests of rice, rice water weevil, *Lissorhoptrus oryzophilus* (Kushal), and fall armyworm, *Spodoptera frugiperda* (J.E. Smith) was explored using mutants with reduced epicuticular wax in comparison with wild-type plants. When the female weevils were given a choice for oviposition, the number of larvae emerging from the mutants was higher than the ones that emerged from a wild type that has the normal wax amount. In addition, the weight gained by the fall army worm larvae were found to be higher in mutants compared to wild-type plants confirming the role of wax in defense against herbivores (Bernaola et al. 2021).

Shi et al. (2023) recently showed that there are 19 wax compounds in rice leaves and sheaths that include acids, alkanes, aldehydes, and alcohols such as hexacosanoic acid, triacontanal, octacosanal, pentacosane, 1-tetracosanol to name a few. In addition, they also found a strong relationship between soil nitrogen and the age of rice plants, with the wax composition and content. Researchers concluded that as the plants age, wax content increases and can thereby suppress pest attacks as part of physical defense. In addition, nitrogen has an important role in determining the wax composition as the content of acid and alkanes in wax were found to increase under reduced nitrogen levels, suggesting the negative effect of nitrogen on wax content. However, more in-depth research will be required to ascertain what aspects of rice EWs may be influencing the behavior of herbivores, and more importantly, whether rice EW quality and quantity can be altered by herbivory, and herbivore feeding types. More recently, a study of sorghum against sugarcane aphids found that wax components such as α-amyrin and isoarborinone were found to increase in 10-day sorghum plants after aphid infestation (Cardona et al. 2023).

### Trichomes

**Fig. 1:**
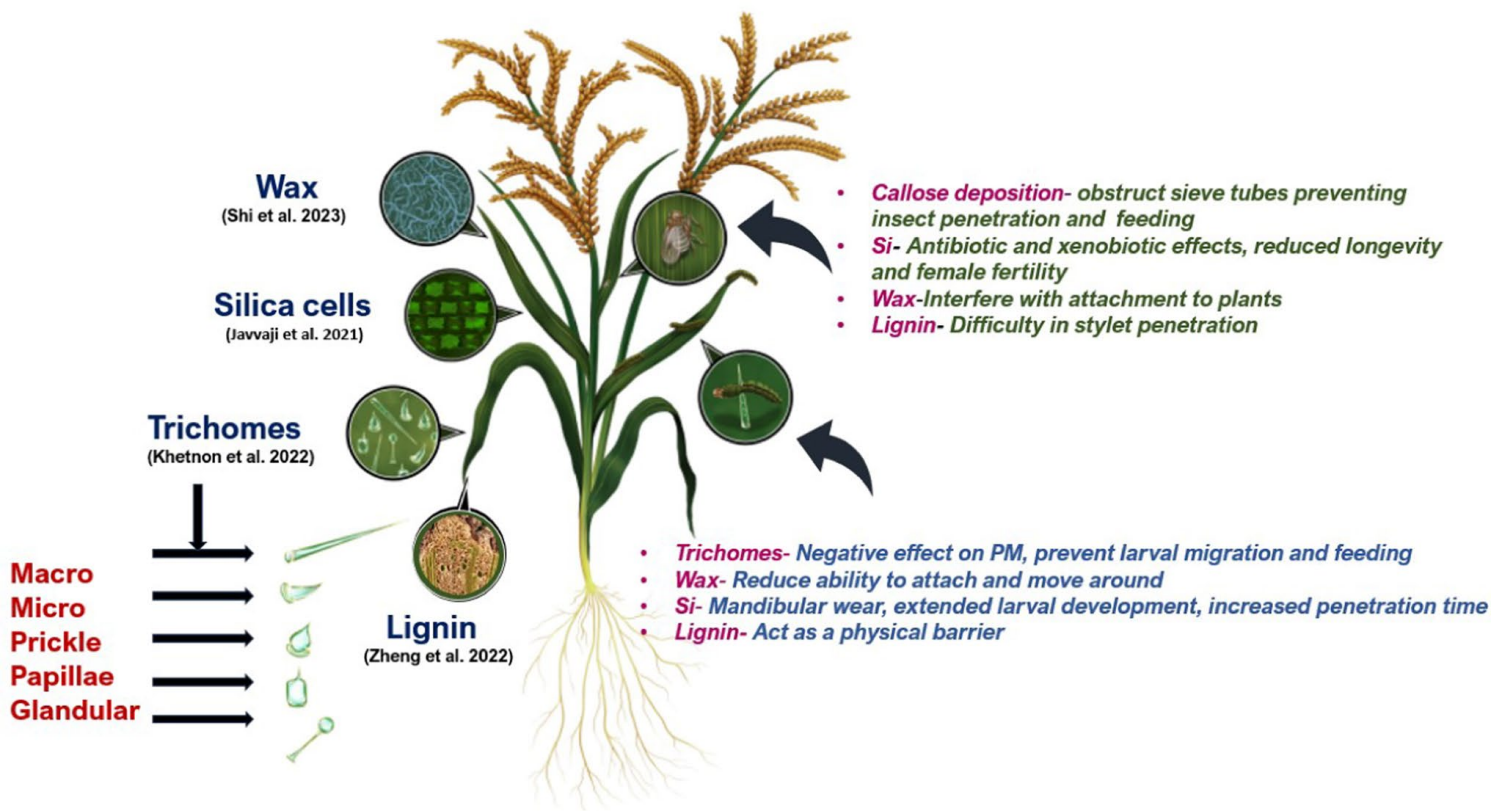
Schematic representation of the physical defense structures in rice and the effects against the major feeding guilds: chewing and sucking herbivores. Wax, trichomes, silica, and callose can be considered as the physical defense mechanisms evolved by plants in response to insect herbivory. Among these, epicuticular wax and trichomes can be considered as surface defense structures, and wax is known as the first line of defense acting as a vigorous barrier against rice insect pests regardless of feeding guilds. Wax in rice restricts the free movement of insect pests resulting in the reduction of mass gain and increased mortality. Trichomes on the other hand penetrate the midgut impeding their feeding. Different types of trichomes are seen in rice such as macro trichomes, micro trichomes, prickle, papillae [non-glandular trichomes], and glandular trichomes. Silica is important in the normal growth and development of rice and has a vital role in defense causing mandibular wear and reducing the longevity and fertility in insect pests. Deposition of callose is another defense mechanism by rice on sucking herbivores such as Brown Plant Hopper, by obstructing the sieve tubes, interfering the stylet penetration

Trichomes in rice have been found to differ in terms of density, length, degree of hardness, growth direction, and form type (Xiao et al. 2017), and can also vary among varieties. In rice, it is commonly observed that the trichome density and their distribution are not uniform. However, a higher trichome density is found on leaves and glumes, compared to other plant parts. Among leaves, there are three different types of trichomes commonly observed—micro, macro, and glandular hairs. Macro hairs are observed in silica cells; whereas, micro and glandular hairs are found on stomatal cells or beside the motor cells (Li et al. 2010). Khetnon et al. (2022) showed that rice can have four types of non-glandular trichomes: prickle, macro, micro and papillae trichomes (Fig. 1). Characterizing its distribution over different varieties, Viz and Pacada (2022) investigated the trichome profiling of the traditional rice varieties and found variations among them and found a specific pattern in their density and dispersion and concluded, trichomes were densest (4.56–5.46/mm^2^) and mostly dispersed (49.09–50.53%) in the apical zone of the leaf surface, and least dense (2.62–2.83/mm^2^) and rarely distributed (16.44–18.81%) in the basal zone. This discovered pattern of diminishing trichome density and distribution on the adaxial leaf blade surface from the apical zone to the base zone can be potentially used for developing varieties that might help defense against rice stem borers through enhanced structural defenses. There is also variation in the orientation of trichomes, wherein they are either erect or recumbent (Viz and Pacada 2022) Considering the two types, more erect hairs can potentially act as a better barrier against pests like stem borers (yellow stem borer and white stem borer) to deter oviposition (as observed in other species (Levin 1973; Hawthorne et al. 1992; Juvik et al. 1994; Resende et al. 2006; Murungi et al. 2016), and thus, decrease infestation, although empirical evidence is currently lacking.

In Punithavalli et al. (2013) examined the function of rice trichomes against the rice leaf folder, *C. medinalis,* and demonstrated their significance in preventing larval migration and the challenge of the larvae for creating folds for feeding within. Sandhu and Sarao (2021) reported that the population of nymphs and adults of BPH were much lower in genotypes with longer and denser trichomes than in susceptible genotypes such as TN1. However, Khetnon et al. (2022) claimed that the physical defense by trichomes was not effective against BPH because trichomes were not tough enough to prevent colonization by the second and third instars. According to Karban et al. (2002), the removal of silicified non-glandular trichomes in rice increased the frequency of damage to leaf sections including the basal region, which was not preferred by the herbivores in the presence of unidirectional structures. Researchers concluded that unidirectional structures such as trichomes are involved in directing the herbivore movement and, hence, can be adopted in plant breeding programs against insect pests. In addition, Andama et al. (2020) demonstrated the vital role of trichomes in imparting defense against insect pests indiscriminately affecting both generalists as well as specialists. Nerica, a new interspecific hybrid rice variety used in Africa was found to be more infested when compared to the local Japanese variety, Nipponbare even when the former has a strong volatile profile. In connection with this, they found the presence of non-silicified glandular trichomes in Nipponbare compared to Nerica, and the larvae fed on Nipponbare showed a higher mortality rate. Larvae fed on Nipponbare on dissection were observed with prevalent punctures and holes in the midgut as well as found strong undigested trichomes from the frass stressing out the pivotal role of trichomes in defending the herbivory (Andama et al. 2020) as previously observed in other systems (Kariyat et al. 2017).

### Callose deposition

Callose, a β-(1,3)-D-glucan polymer, plays an important role against phloem-feeding insects and pathogens in the plants by depositing callose at the site of the attack to slow down their feeding and spread (Miles 1999). Callose is usually present in sieve plates of phloem, pollen grains, pollen tubes, and pollen mother cells in plants (Stone and Clarke 1992). During feeding by phloem-feeding insects, plants up-regulate genes encoding callose synthase and β-(1,3)-D-glucanases, activating callose synthase genes that initiate the callose synthesis and deposition of callose on the sieve plates of plants (Will and van Bel 2006; Kempema et al. 2007; Louis et al. 2012; Mondal et al. 2018; Varsani et al. 2019). The callose deposited on the sieve plates obstructs the sieve tubes and prevents the insect's penetration and feeding. However, β-(1,3)-D-glucanase can degrade the callose permitting phloem feeders to continue feeding significantly more in susceptible plants compared to resistant plants resulting in heavy damage to the susceptible rice plants (Hao et al. 2008). Callose deposition is also induced by the stress hormone, Abscisic acid (ABA) where Liu (2017a), explained the exogenous application of ABA can reduce the activity of the hydrolyzing enzymes and thus callose synthesis is unaffected. Here, the ABA showed a positive impact on rice by increasing its resistance to BPH, reducing its fecundity, and eventually acting as a barrier against the piercing and sucking insect pests; however, the exact mechanism behind this remains unresolved.

### Lignin

Lignin is present in the cell wall of plants and in rice, lignin content varies from 10 to 14% (Wijayanti et al. 2019) depending upon the varieties and has an important role in preventing herbivory. Lignin accumulation in cell wall helps in imparting resistance to BPH (Jannoey et al. 2015; Guo et al. 2018; He et al. 2020) and according to Zheng et al. (2020), in addition to the callose deposition in response to BPH attack, lignin helps in providing mechanical rigidity to the rice sheaths, making it difficult for them to penetrate their stylets. This was in line with the findings of Wijayanti et al. (2019) who reported that having higher lignin and cellulose content tends to resist BPH attack. According to Zhang et al. (2022), regulated genes related to BPH attack are engaged in the synthesis of lignin and flavonoids, which demonstrates the post herbivory induction of lignin and its function as a physical barrier against important sucking pests in rice. Although the majority of research on lignin production is related to sucking pests, there have been few studies on chewing insect pests in rice, such as leaf rollers. Tianpei et al. (2015) showed that treating rice with an insect-specific peptide LqhIT2 was found to increase the accumulation of lignin resulting in imparting resistance to leaf roller. In addition, fertilization also has a strong impact on the lignin accumulation in rice, especially nitrogen fertilizers. A study conducted by Zheng et al. (2021), reported that low nitrate content (with a concentration of 0.3 mM KNO_3_) can increase the lignin concentration as well as other defense compounds such as flavonoids, phenolic acids, and saccharides, which have also been implicated in imparting resistance against insect pests—especially flavonoids (Kariyat et al. 2019; Tayal et al. 2020; Singh et al. 2021). Clearly, lignin helps in acting as a passive barrier as well as initiating other defenses in plants, in this case, against the striped stem borer, *C. suppressalis*. Collectively, these studies show the importance of lignin as an important component of physical defenses in rice.

### Silicon

Silicon (Si) is the most abundant element in the earth's crust and has a crucial role in providing resistance against insect pests including chewing (florivores and borers) and sucking herbivores (phloem and xylem feeders). Si has direct effects on reducing the herbivore performance and indirect effects on the attraction of natural enemies by delaying the overall herbivore establishment (Reynolds et al. 2009). There are different forms of silica cells, such as butterfly-shaped in rice and maize, and oval-shaped in wheat (Alhousari and Greger 2018), and their cell differentiation is mediated by the phytohormone, jasmonic acid (JA). Si increases plant resistance by depositing silica, especially in opaline phytoliths. Phytoliths are the minute amorphous silica structures that are formed by the precipitation and polymerization of silica within and between plant cells (Piperno 2006). This helps in increasing the hardness and abrasiveness of plant tissues followed by reducing the digestibility (Kaufman et al. 1985; Salim and Saxena 1992; Panda and Khush 1995; Ma et al. 2001; Massey et al. 2006; Massey and Hartley2008), and causing mandibular wear in herbivores (Djamin and Pathak 1967; Dravé and Laugé 1978; Ramachandran and Khan 1991) and the degree of wear has a positive relation with the silicon concentration (Massey and Hartley 2008). Molting of mandibles occurs in each instar and, hence, mandibular wear cannot be only considered for the negative impacts in herbivores; moreover, destructive effects on silica on the digestive tract will add the impact as the digestive tract never molts reducing the nitrogen absorption leading to drastic reduction in the relative growth rate (Massey and Hartley 2008) and survival rate of caterpillars (Han et al. 2016).

Rice can normally absorb 300–700 kg/ha Si (Snyder et al. 1986) during different growth stages and hence has an important role in imparting resistance against herbivory (Mitani et al. 2005) (Table 2). The genetic evidence of the role of Si in imparting resistance was proved by Nakata et al. (2008), where the damage by the lepidopteran pests, rice leaf folder, *C. medinalis,* and rice green caterpillar, *Naranga aenescens* was more pronounced in Si-impaired *low silicon rice 1* (*lsil*) mutant than the wild-type plants. Quite a few studies have demonstrated the role of Si in reducing the infestation of chewing herbivores of rice: rice stem borer *Chilo suppressalis* (Sasamoto 1958; Djamin and Pathak 1967; Drav´e and Laug´e 1978) and leaf folder larvae, *C. medinalis* (Hanifa et al. 1974; Ramachandran and Khan 1991). Studies have also shown that the presence of Si can increase the penetration time of insects to plants as in Asiatic rice borer, *C.suppressalis* (Hou and Han 2010). Subbarao and Perraju (1976) reported a significant reduction in insect infestation along with increased Si plant uptake with the soil drench of potassium silicate (K_2_SiO_3_) in rice against *S. incertulas*. In addition, Si can trigger the plants to produce, escalate or alter HIPVs (Herbivore Induced Plant Volatiles) that can either repel insect pests or attract natural enemies (Kvedaras et al. 2010). Liu et al. (2017b) reported a significant increase in the attraction of parasitoids, *Trathala flavo-orbitalis* and *Microplitis *sp. to Si-treated plants after the infestation of rice leaf folder. There was a variation in the HIPVs produced such as hexanal 2-ethyl, α-bergamotene, -β-sesquiophellandrene and cedrol, in infested Si-treated plants compared to non-treated plants, and the signaling pathway responsible for inducing resistance is JA. Similarly, Lu et al. (2015) reported the improved resistance of Si-treated plants against rice water weevil which is mediated by JA signaling. In short, Silica has a crucial role in rice in determining the degree of resistance toward defoliators.

**Table 2:** Si-mediated plant defense against rice pests

Insect species	Resistance mechanism	Reference
Rice leaf folder *Cnaphalocrocis medinalis*	Extended larval development and reduced weight gain in third instars, larval survival, pupation rate, and pupal weight, reduced ovipositional preference, induced defense by HIPV production	Javvaji et al. (2021), Han et al. (2015, 2016, 2017)
	Priming defense-related enzymes	Ye et al. (2013)
Asiatic rice borer *Chilo suppressalis*	Decreased and prolonged borer penetration, reduction in weight gain, and stem damage	Hou and Han (2010)
Yellow stem borer, *Scirpophaga incertulas*	Decrease in weight, Mandibular wear, rupture of the peritrophic membrane	Jeer et al. (2016)
Sugarcane borer, *Diatraea saccharalis*	Reduced boring and relative growth rate	Sidhu et al. (2013)
Brown planthopper *Nilaparvata lugens*	Modulation of callose deposition	Hao et al. (2008), Yang et al. (2018)
	Antibiotic and xenobiotic effects targeting insect physiological functions	He et al. (2015), Hou et al. (2017)
	Impair sucking behavior by prolonging the stylet pathway, reduced feeding preference and overall population growth rate	Yang et al. (2017b)
White backed plant hopper, *Sogatella furcifera*	Reduces adult longevity and female fertility	Salim and Saxena (1992), Yang et al. (2014)

In addition to defoliators, Silica also has a strong negative impact on piercing and sucking pests in rice. In phloem feeders such as BPH, Yang et al. (2017a) reported a significant decrease in the infestation in Si-treated rice. Here, Si deposition prolonged the time in the stylet pathway, thereby shortening the duration of phloem puncture and ingestion. Si deposition increased the hardiness and toughness of plant tissues, making BPH difficult to penetrate the tissues by the elongation of non-probing and stylet pathway activities. Another reason for this is the increased deposition of callose that obstructs the mass flow of the phloem, blocking the phloem sap leakage (Hao et al. 2008). Si also plays a role in the biochemical and physiological changes in plants by decelerating the increase in malondialdehyde (MDA) concentrations that attenuate the stress from BPH attack. In addition, Si can reduce the palatability of plant tissues through the activities of polyphenol oxidase and peroxidase phenylalanine ammonia lyase that catalyze the oxidation of phenols to quinones (Yang et al. 2017a). Hence, these studies conclusively demonstrate that silicate supplements can stimulate a variety of plant defense mechanisms against both chewing and phloem-feeding insect invaders by modifying plant secondary metabolites and antioxidant defense mechanisms.

## Genes involved in surface defenses in rice

In rice, there are three important genes that are identified to be involved in trichome formation. *GLABROUS RICE 1* (*GLR1*, otherwise known as *WUSCHEL-LIKE HOMEOBOX 3B [OsWOX3B], DEGENERATIVE PALEA [DEP]*, and *NUDA*—a gene encoded by a WUSCHEL (WUS)-like homeodomain protein that assists in trichome formation. Another is *HAIRY LEAF 6 (HL6)* which interacts with *OsWOX3B*, encoding APETALA2/ETHYLENE RESPONSE FACTOR-type transcription factor initiating the trichome elongation and formation. *SQUAMOSA PROMOTER BINDING PROTEIN-LIKE10 (OsSPL10)*, is the recently identified gene also found to be responsible for trichome production. Sun et al (2017), reported that *HL6* interacts with *OsWOX3B* to form a protein complex that increases the binding of *HL6* with an auxin-related gene, OsYUCCA5 leading to the development of trichomes. In a recent study, Li et al. (2021) confirmed the role of *OsSPL10*, by disrupting the gene by genome editing which resulted in the reduction of trichome density and length. Another defense is callose synthesis which is controlled by 2 UDP-glucose pyrophosphorylase (UGPase) genes ((*UGP1* and *UGP2*) and 10 glucan synthase-like (GSL) genes (Chen et al. 2006; Shi et al. 2015)) in rice. Ke et al. (2019) using a mutant *pex1* (Leucine-rich repeat extensin-like protein), found a higher lignin content and increased expression of lignin biosynthesis genes in this mutant compared to the wild types. This is because the mutant was formed by the ectopic expression of a leucine-rich repeat extension-like gene, *OsPEX1* and they observed the reduced lignin content in *OsPEX1* suppressed plants confirming the role of *OsPEX1* gene in lignin biosynthesis. According to Kawasaki et al. (2006), OsRac1, belonging to GTPases is the enzyme responsible for regulating the lignin deposition in the cells as a defense reaction and this is through the regulation of NADPH oxidase and the activities of cinnamoyl-CoA reductase 1 (OsCCR1) that is an effector of OsRac1. Similarly, another protein, named GLPs (Germin-like proteins), is also responsible for altering the lignin synthesis genes and helps regulate the lignin accumulation in the cell walls (Shanguan et al. 2023). Needless to say, more comprehensive studies incorporating genomics and transcriptomics are needed to elucidate the genetic and molecular networks involved in the physical defense mechanisms in rice.

## Conclusion and future perspectives

As discussed in this mini review, plant structural traits such as trichomes, epicuticular wax, silicon, callose deposition, and lignin have crucial roles in reducing insect herbivory in rice. However, these structural traits are not explored and not well understood, especially in the context of rice-herbivore interactions, when compared to their dicot counterparts. Insect herbivory and pesticide resistance development are serious concerns, and are due to the indiscriminate use of chemicals and pesticide residue that affects the quantity and quality of rice, pre and post harvesting. In addition to resistance against insect pests, surface defenses such as trichomes have a crucial role in protecting plants from changing climatic conditions such as extreme temperatures, water stress, and UV irradiation (Hu et al. 2013; Lan et al. 2019). There is a critical need in elucidating the role of each physical barrier in terms of type, structure, mode of action in controlling the invading pests, which has consequences for determining pest control strategies and the development of resistant traits—thus reducing the build-up of chemicals in the field. Just in the case of trichomes, the morphological diversity at genus and species levels in dicots have been found to have functional consequences (Watts et al. 2023) and clearly with current advances in microscopy and imaging techniques, this can be better resolved. The need for more rice and rice-based products is a global concern and hence, rice production can be improved by exploring the mechanisms underlying plant–herbivore interactions and unveiling how rice plants can use their own defense mechanism to cope up this constant and continuous biotic stress. Research on genetic and molecular mechanisms of physical defenses and identifying the genes responsible for the defense mechanisms can be utilized in plant breeding programs to improve the yield minimizing the environmental impacts, thereby managing pests in a sustainable way.

## Data Availability

Not applicable.

## Abbreviations

HIPVs: Herbivore-induced plant volatiles
Si: Silicon
BPH: Brown plant hopper
JA: Jasmonic acid
EW: Epicuticular wax
